# Iron Promotes the Retention of Terrigenous Dissolved Organic Matter in Subtidal Permeable Sediments

**DOI:** 10.1021/acs.est.3c09531

**Published:** 2024-04-01

**Authors:** Zhe Zhou, Hannelore Waska, Susann Henkel, Thorsten Dittmar, Sabine Kasten, Moritz Holtappels

**Affiliations:** † Alfred Wegener Institute Helmholtz Centre for Polar and Marine Research, Bremerhaven 27570, Germany; ‡ State Key Laboratory of Marine Geology, Tongji University, Shanghai 200092, China; § Institute for Chemistry and Biology of the Marine Environment (ICBM), School of Mathematics and Science, Carl von Ossietzky Universität Oldenburg, Oldenburg 26129, Germany; ∥ Helmholtz Institute for Functional Marine Biodiversity, University of Oldenburg, Oldenburg 26129, Germany; ⊥ MARUM - Center for Marine Environmental Sciences, University of Bremen, Bremen 28359, Germany; # Faculty of Geosciences, University of Bremen, Bremen 28359, Germany

**Keywords:** permeable sediments, dissolved organic matter, iron, redox cycling, terrestrial input

## Abstract

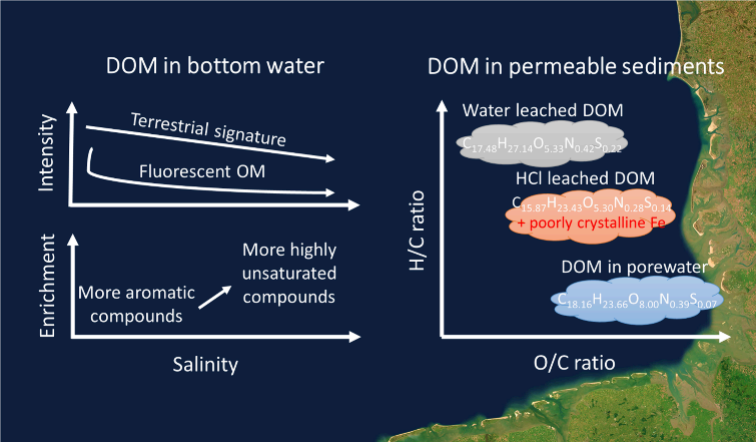

Marine permeable sediments are important sites for organic matter turnover in the coastal ocean. However, little is known about their role in trapping dissolved organic matter (DOM). Here, we examined DOM abundance and molecular compositions (9804 formulas identified) in subtidal permeable sediments along a near- to offshore gradient in the German North Sea. With the salinity increasing from 30.1 to 34.6 PSU, the DOM composition in bottom water shifts from relatively higher abundances of aromatic compounds to more highly unsaturated compounds. In the bulk sediment, DOM leached by ultrapure water (UPW) from the solid phase is 54 ± 20 times more abundant than DOM in porewater, with higher H/C ratios and a more terrigenous signature. With 0.5 M HCl, the amount of leached DOM (enriched in aromatic and oxygen-rich compounds) is doubled compared to UPW, mainly due to the dissolution of poorly crystalline Fe phases (e.g., ferrihydrite and Fe monosulfides). This suggests that poorly crystalline Fe phases promote DOM retention in permeable sediments, preferentially terrigenous, and aromatic fractions. Given the intense filtration of seawater through the permeable sediments, we posit that Fe can serve as an important intermediate storage for terrigenous organic matter and potentially accelerate organic matter burial in the coastal ocean.

## Abstract 2

Permeable sediments are efficient sites for dissolved organic matter (DOM) turnover. Our study reports DOM molecular compositions along a near-shore to off-shore transect and explores the role of poorly crystalline iron for the retention of terrigenous DOM in subtidal permeable sediments.

## Introduction

Marine dissolved organic matter (DOM) is one of the earth’s major carbon reservoirs and, thus, the subject of intense research.^1−3^ Ocean sediments provide a large interface and may act as both source and sink for DOM.^4−6^ Permeable sandy sediments that cover more than 50% of continental shelves are efficient sites for the turnover of organic matter in particulate (POM) as well as dissolved forms.^7−11^ Advective flow in permeable sands drives seawater filtration (in hundred L/m^2^/day) and the retention of POM and DOM in the sediments.^12^ DOM in permeable sediments can be advected to greater sediment depths and across various redox zones, where it can react with the solid phase and attached biota.^13−15^ Molecular compositions of DOM (e.g., elemental ratios, fluorescent OM, and aromatic contents), both in the aqueous phase and in the exchangeable solid phase, can be useful indicators to differentiate DOM sources and assess DOM cycling in the coastal ocean.^16,17^ So far, our knowledge about molecular compositions of DOM in marine subtidal permeable sediments is very limited.^18^^,^^19^

Coastal sediments receive a mixture of organic matter (OM) from various marine, terrestrial (e.g., river and groundwater), and anthropogenic sources.^20−24^ The proportion of OM originating from different sources is controlled by regional geological settings, rates of erosion and weathering, physical transport and mixing, biotic/abiotic transformation, and decomposition processes along the land–ocean transition zone.^24,25^ Moving away from the coasts, terrigenous DOM continuously declines in abundance and is replaced by marine DOM, which is mainly derived from plankton and comparatively more biodegradable.^24,26,27^ OM degradation in permeable sediments is facilitated by oxygen supply via porewater advection.^7,8,28^ As redox conditions change with sediment depth, OM remineralization continues with a shift in terminal electron acceptors from oxygen to nitrate, manganese and iron oxides, and sulfate—although at lower rates for anaerobic compared to aerobic degradation.^29−32^ The degradation of OM is expected to follow an intrinsic reactivity continuum,^33^ with less bioavailable, less saturated, and more aromatic compounds enriched along the redox gradients.^34−37^ In subtidal permeable sediments, porewater advection can cause frequent variations of redox conditions and mass exchanges,^10,38,39^ thus disrupting the horizontal redox zonation and degradation pathways.^40,41^

Porewater advection in sandy sediments results in filtration and retention of DOM and OM exchange between the solid and aqueous phases. In marine surface sediments, about 22% of total OM are associated with Fe phases (extracted by citrate-dithionite).^42^ Under variable redox conditions that prevail in sandy surface sediments, the interactions between DOM and Fe become much more complex.^43^ DOM can be preferentially adsorbed on the surface of the Fe(III) oxyhydroxides. DOM fractions that have comparatively higher molecular weight, less saturated, and more oxygen-rich formulas are preferentially scavenged.^44−46^ Under reducing conditions, adsorbed DOM can be partially released back into the solution due to the reductive dissolution of Fe(III) oxyhydroxides.^42,45^ During the reoxidation process, DOM can be recaptured by Fe^2+^ oxidation and precipitation,^43^ and reactive oxygen species that form during Fe^2+^ oxidation may further alter the molecular composition of DOM.^47−49^ The effects of DOM–Fe interactions on DOM cycling in sands, however, are not fully understood.

The molecular composition of DOM in different marine compartments, including bottom water (seawater directly overlying sediments), porewater, and fractions exchangeable with the sedimentary solid phase, provides important information regarding the sources and turnover of DOM in coastal sediments. Here, we explore the interactions between DOM and Fe in sandy sediments with dynamic redox conditions, via quantitative and qualitative comparisons of DOM in porewater, DOM leached by ultrapure water (loosely adsorbed fraction), and DOM leached by 0.5 M HCl (loosely adsorbed and poorly crystalline Fe preserved fractions). We collected permeable sandy sediments at seven stations along a nearshore to offshore transect in the North Sea (Germany). The molecular composition of DOM in bottom water and porewater and DOM adsorbed onto different solid phases was qualitatively analyzed with electrospray ionization Fourier transform ion cyclotron resonance mass spectrometry (ESI-FT-ICR-MS). This untargeted approach provides semiquantitative information on several thousand molecular formulas of DOM constituents simultaneously. Our main questions were (1) How does the DOM molecular composition vary along the near- to offshore gradient? (2) What is the quantity and quality of DOM associated with poorly crystalline Fe in the bulk of permeable sediments? (3) What is the potential role of coastal permeable sediments in the retention of terrigenous DOM?

## Experimental Section

### Study Area and Core Collection

**Figure 1:**
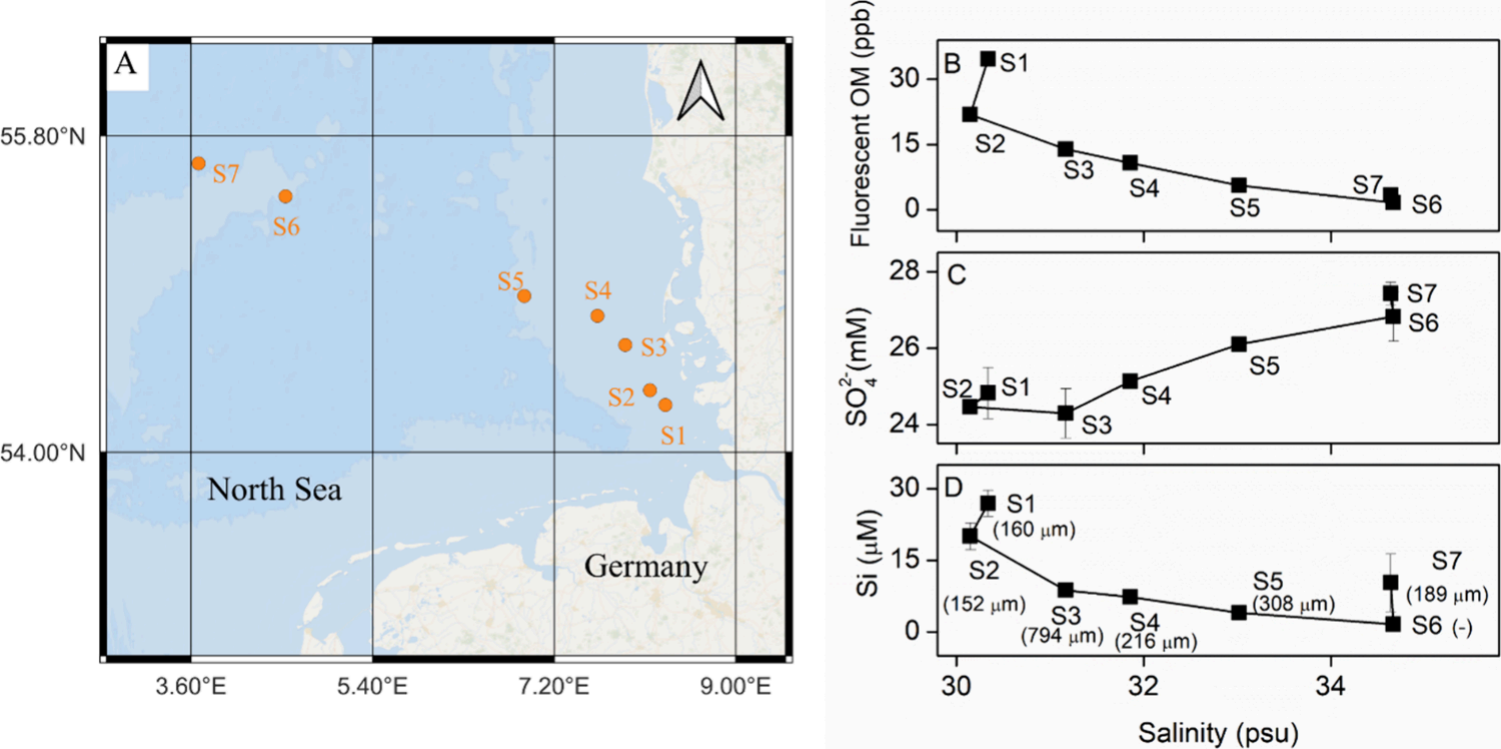
(A) Map of sampling stations in the German North Sea; (B) abundance of “terrigenous and aquatic humic-like” fluorescent OM (FDOM, details in the experiment description), (C) SO_4_^2–^ concentrations, and (D) silicate concentrations over salinity in the bottom water of each station. The average grain size (in μm) for each station is added in (D).

Samples were collected during a cruise with RV Heincke (HE582) from August 23 to September 5, 2021.^50^ The study area was located in the German Bight of the North Sea with water depths ranging from 8.8 to 36 m (Figure 1A). We visited 7 stations characterized by sandy sediments with different mean grain sizes. The stations formed a transect from shallow coastal waters near the Elbe estuary to deeper offshore waters at the Dogger Bank. At each station, the work program started with initial multibeam surveys and grab samples to characterize seabed topography (e.g., large bedforms) and sediment properties, followed by the deployment of two automated benthic observatories for in situ measurements of bottom waters and sediments. Afterward, undisturbed sediment cores were retrieved with a multiple corer (MUC, Oktopus Kiel) equipped with acrylic tubes (inner diameter of 10 cm). These cores were used to conduct bottom water, porewater, and solid-phase sampling and extractions. Regarding the sediments, we focused on the sediment–water interface (from a depth of 1–6 cm below the sediment surface).

### Aqueous-Phase Sampling

The subsequent sampling of bottom water and porewater and the extractions of sediment were conducted immediately on board. All syringes, pipet tips, falcon tubes, and containers were prewashed with 1 M HCl (p.a.) and ultrapure water, and their blanks were collected and tested for quality control. Polyethylene (PE) syringes and rhizons (pore size 0.15 μm) were used to collect the overlying bottom water (∼2 cm above sediments) and porewaters from sediment depths of 1–2 and 5–6 cm, respectively, according to the procedure described previously.^51^ First, the overlying bottom water was sampled, which was then removed from the cores before the porewater was collected. FDOM (excitation center-wavelength 375 nm, emission wavelength >420 nm, “terrigenous and aquatic humic-like”)^52^ and the pH of samples were measured on board using a handheld fluorometer (Aquafluor, Turner Instruments) and a portable pH meter (WTW), respectively. The bottom water and porewater samples were split into aliquots and stored for further analysis in home laboratories. Samples for DOC and molecular DOM analyses (∼15 mL) were stored in acid-washed high-density polyethylene (HDPE) bottles, preserved via acidification with double-distilled HCl to pH 2, and stored at 4 °C in the dark. About 4 mL samples were rapidly frozen at −20 °C in falcon tubes for the determination of nutrients (NO_3_^–^, NH_4_^+^, PO_4_^3+^, and silicate). Samples for the analysis of dissolved inorganic carbon (DIC) (1.5 mL) were filled without headspace into airtight glass bottles, conserved with HgCl_2_, and stored at 4 °C. DIC and nutrients were analyzed with a QuAAtro continuous segmented flow analyzer equipped with different modules (Seal Analytical). The samples (1 mL) for inductively coupled plasma optical emission spectrometry (ICP-OES, Thermo Elemental) were acidified with double-distilled HCl (pH < 2) and stored at 4 °C. Dissolved Mn, Fe, P, Si, Ba, As, Al, Cu, Li, and Mo were measured in 1:10 dilutions with Y as an internal standard to correct for different ionic strengths. Residual unacidified samples were used for the analysis of SO_4_^2–^ and Cl^–^ (1:50 dilution with ultrapure water) by ion chromatography (Metrohm, Compact IC Flex 930).

### Sediment Leachates

The cores were sacrificed immediately after porewater extraction. Sediments from depths 1–2 and 5–6 cm were sampled on board, and 1 cm^3^ wet sediments (1.5–1.9 g) from each depth were leached separately with 40 mL ultrapure water (hereafter referred to as “UPW leachate”) and 0.5 M double-distilled HCl (hereafter referred to as “HCl leachate”). UPW is expected to leach the loosely adsorbed DOM, while 0.5 M HCl dissolves poorly crystalline Fe oxyhydroxides as well as Fe monosulfides and is expected to leach excess, iron-bound DOM in addition to the loosely adsorbed fraction. The difference between DOM abundance in HCl and UPW leachate was calculated as the amount of DOM associated with poorly crystalline Fe. The sediment leaching was carried out using prewashed polypropylene (PP) falcon tubes on an end-overend rotator for over 1 h in the dark. Then, the solutions were filtered through 0.22 μm poly(ether sulfone) (PES) filters. Process blanks for UPW, 0.5 M HCl, and filters were collected at the beginning and the end of the expedition and demonstrated that DOC concentrations in these process blanks were less than 5% of sample DOC concentrations. The samples for DOC, molecular DOM, and ICP-OES analyses were preserved as described earlier. The leached Fe (by 0.5 M HCl) was determined directly on board with the revised ferrozine method to differentiate Fe(II) and Fe(III).^53^ Furthermore, sediments from each station were stored at 4 °C and characterized with a laser diffraction particle size analyzer (Beckman Coulter LS 200). Subsamples of the wet sediments from each station were freeze-dried, the percentage of water content was calculated based on the weight changes, and the total organic carbon (TOC) was measured with a TOC analyzer equipped with a halogen scrubber (Elementar Vario EL III).

### Molecular Analysis of DOM and Data Processing

For all sample types, aliquots of 5 mL were transferred into precombusted DOC autosampler vials and filled up to 10 mL with ultrapure water acidified to pH 2, or ultrapure water for HCl leachates. DOC was determined with a Shimadzu TOC-VCPH analyzer, and the results were verified with the help of the deep-sea Atlantic reference material (Hansell Lab, FL, USA). The precision and trueness of the DOC measurements were better than 5%. The pH of the remainder of the samples was adjusted to 2 with HCl or NaOH (HCl samples), and desalted and concentrated via solid-phase extraction (SPE) using Agilent BOND ELUT PPL 100 mg cartridges following previous recommendations.^54^ After passing the samples through the cartridges, the SPE sorbents were repeatedly rinsed with 0.01 M HCl (p.a.), dried with Ar gas, and eluted into precombusted amber glass vials with HPLC-grade methanol. Two process blanks with ultrapure water (adjusted to pH 2 with HCl) were extracted together with the samples. On average, sample volumes were 27.7 ± 11.5 mL, and methanol extract volumes were 0.73 ± 0.03 mL. For the determination of DOC extraction efficiencies, aliquots of 300 μL of each extract were transferred into DOC autosampler vials, dried in an oven overnight at 50 °C, and redissolved in 0.01 M HCl (p.a.). The SPE-DOC concentrations in the extracts were then converted into SPE-DOC concentrations in bottom water under consideration of the different extraction volumes and dilution factors. Extraction efficiency was ultimately defined as the contribution of SPE-DOC to the original bulk DOC concentrations. Overall, bottom water samples had SPE efficiencies of 46.7 ± 7.1%, porewater of 43.9 ± 8.0%, UPW leachates of 19.6 ± 6.9%, and HCl leachates of 10.4 ± 3.1%. SPE efficiencies of UPW and HCl leachates were relatively low. Assuming that SPE under the same conditions targets the same compound groups, lower SPE-DOM recoveries presumably lead to more molecular uniformity among environmentally different samples. Since our results below demonstrate, all three sample groups (bottom water and porewater, UPW leachate, and HCl leachate) showed distinct molecular signatures, indicating that environmental differences were still preserved. Nevertheless, we suggest that a thorough method re-evaluation is advisable in future studies to accommodate the chemical differences between water-column DOM and exchangeable DOM from sediments.

After SPE, the methanol extracts were adjusted to a DOC concentration of 2.5 ppm with methanol (MS grade) and ultrapure water to reach a ratio of 1:1, filtered through 0.2 μm PTFE filters, and analyzed in negative ionization mode on a 15 T Fourier transform ion cyclotron resonance mass spectrometer (FT-ICR-MS, Bruker solariX XR), equipped with an electrospray ionization (ESI) source and a HyStar Autoanalyzer. The open tool ICBM-OCEAN was used for data processing and molecular formula assignment.^55^ The details about the measurement and the data processing can be found in the Supporting Information. After molecular formula assignment and data postprocessing (i.e., blank and noise removal, replicate correction, and normalization), we calculated several indices from the molecular formula data for each sample, such as the molecular lability boundary index (MLB_L_),^56^ the degradation index (*I*_DEG_),^57^ the bioproductivity index (*I*_bioprod_),^58^ the terrestrial index (*I*_Terr_),^59^ and the aromatic index (AI_mod_).^60^ More detailed information can be found in Table S7. In addition, we grouped the thousands of detected molecular formulas into molecular compound classes via ICBM-OCEAN. The relative proportion of each compound group was weighted by normalized FT-ICR-MS signal intensities in each sample. A principal coordinate analysis (PCoA) was conducted with the vegan package^61^ using the Bray–Curtis dissimilarity of the DOM composition of all samples (i.e., molecular formulas and relative signal intensities). Comparisons of molecular formula abundances between sample groups, for example, UPW leachate samples and HCl leachate samples were done with pairwise Wilcoxon rank-sum tests (*p* < 0.05). Hereby, we used the relative signal intensity of each molecular formula as an indicator of abundance in the respective sample. Sample distributions based on the DOM molecular composition were displayed in the form of a biplot. Correlations between metadata such as DOM molecular indices and geochemical characteristics and the two main PCoA coordinates were calculated with the envfit function and superimposed onto the biplot. Furthermore, Spearman’s rank correlations (*p* < 0.05, ρ ≥ 0.5 or ≤ −0.5) were calculated between relative intensities of individual molecular formulas and environmental parameters (e.g., dissolved iron concentrations). Comparisons of molecular formula abundances between sample groups, for example, UPW leachate samples and HCl leachate samples were done with pairwise Wilcoxon rank-sum tests (*p* < 0.05). Hereby, we used the relative signal intensity of each molecular formula as an indicator of abundance in the respective sample.

## Results

### Near- to OffShore Transect

The seafloor at stations S1, S2, S4, and S7 was mainly (53–68%) covered with fine sand (Table S1). At station S3, coarse to very coarse sand composed 95% of the sediment. At station S5, medium sand is composed of 70% of the sediments. Only in the cores collected from station S1, burrowing macrofauna (sea anemones, starfish, etc.) were visible. TOC contents (in % dry weight) of the sediments were inversely related to average grain sizes, with 0.12% at stations S1 and S2, 0.04–0.05% at stations S4, S6, and S7, and 0.01% at stations S3 and S5.

Along the sampling transect from near- to offshore, the increase of salinity (from 30.1 to 34.6 PSU) and sulfate concentrations (from 24.8 to 27.4 mM) in the bottom water was coupled with the decrease of FDOM (from 35 to 2 ppb; Figure 1). Dissolved silicate concentrations decreased from 26.9 to 1.6 μM moving from station S1 to station S6 but increased to 10.3 μM at station S7 (Figure 1D). Dissolved phosphorus, nitrate, and DIC concentrations in the bottom water did not show any distinct trend (Figure S1). Nitrate in bottom water was nearly depleted (<1 μM) at all of the stations. The pH of the bottom water varied between 8.1 and 8.2 at stations S1–S6 and decreased to 7.9 at station S7 (Figure S1). In the 0.5 M HCl extraction of the sediments, the concentrations of Fe and Mn were higher in finer sands, whereas no significant near- to off-shore trend was found (Tables S2 and S3).

**Figure 2:**
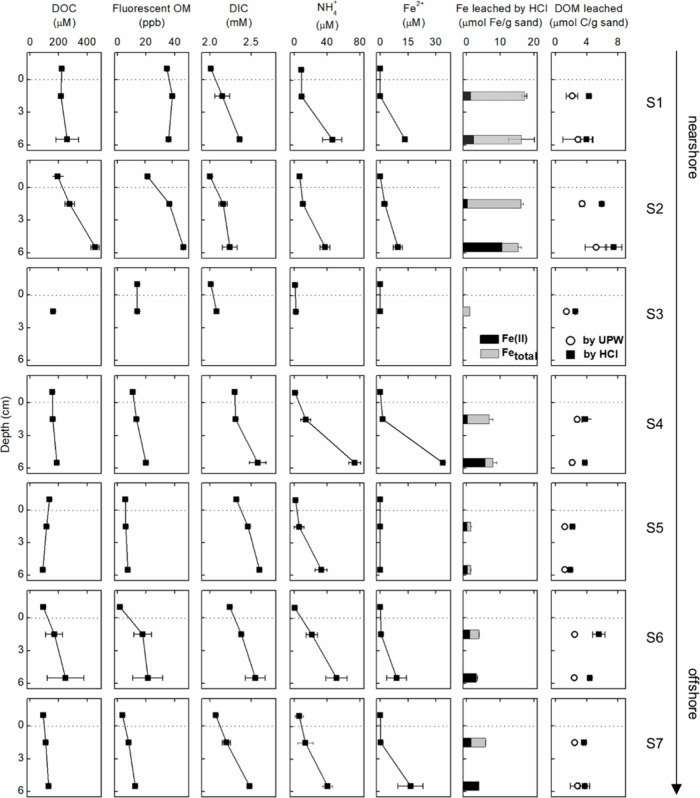
Geochemical parameters of bottom water and porewater as well as of UPW and HCl leachates (1–2 and 5–6 cm) at stations S1 to S7. Duplicate cores and samples were collected and analyzed at each station. Error bars indicate the range of duplicates.

**Figure 3:**
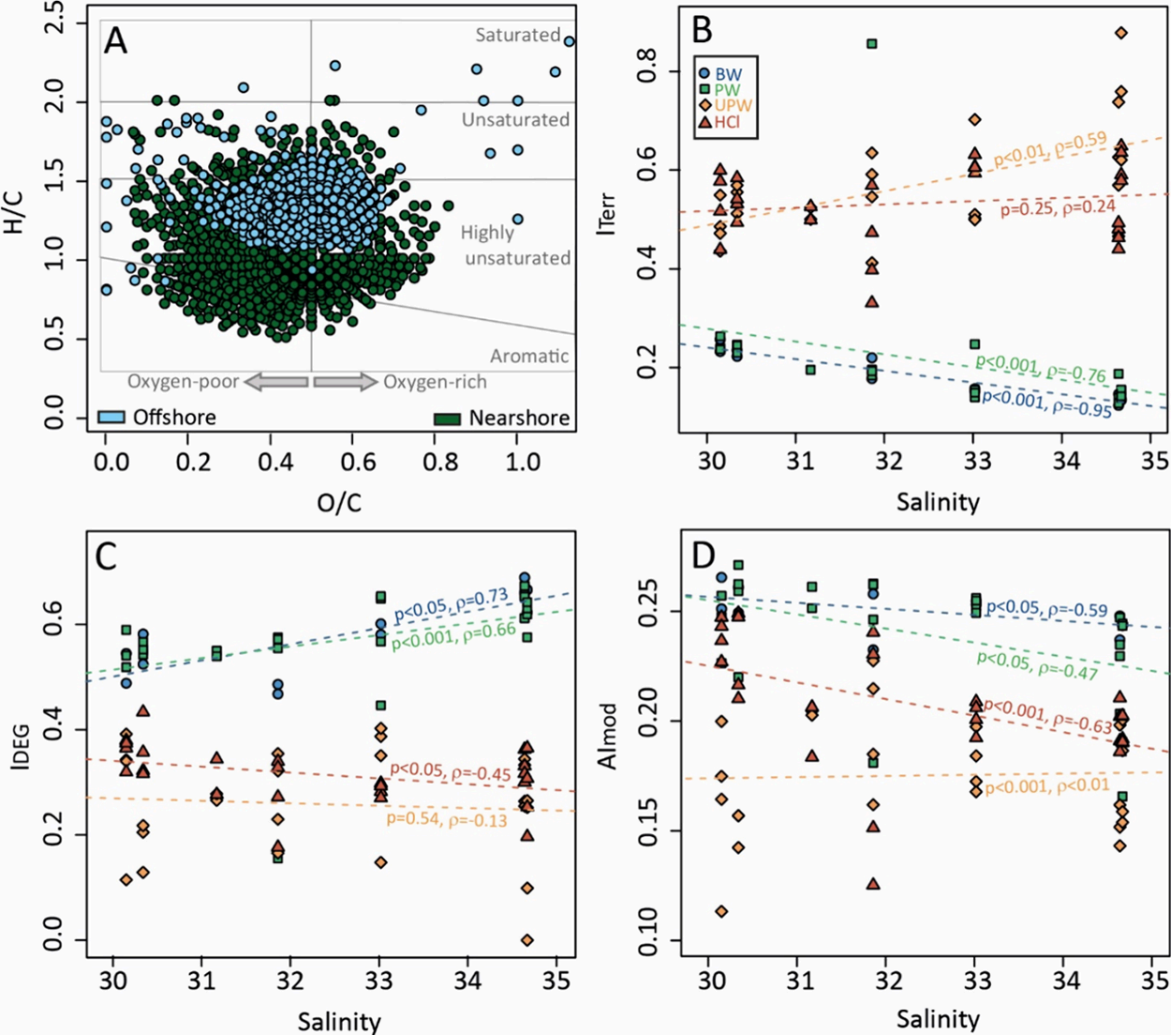
(A) van Krevelen plot of molecular formulas significantly abundant in either near-shore (green, S1 and S2) or offshore (S6 and S7, blue) bottom water and porewater samples. (B–D) Scatter plot between intensity-weighed DOM molecular indices and salinity. Spearman’s p and ρ value for correlation between indices and salinity are shown along the regression lines for each sample type (BW: bottom water; PW: porewater; UPW: ultrapure water leachate; HCl: 0.5 M HCl leachate).

DOC concentrations in bottom water were lower at the offshore stations (Figure 2), and there were clear near- to offshore trends with respect to molecular indices of DOM (Figure 3). From near-shore stations (S1 and S2) to offshore stations (S6 and S7), the percent of unsaturated and highly unsaturated compounds in bottom water and porewater increased from 72 to 99%, while aromatic compounds decreased from 28% to 1% (Table S6). *I*_Terr_ and AI_mod_ of DOM in bottom water and porewater decreased following the salinity gradient (30.1 to 34.6 PSU), while average molecular mass as well as I_DEG_ increased (Figure 3 and Table S4). *I*_Terr_ in the UPW leachate increased along the near- to offshore transect, and AI_mod_ in the HCl leachate decreased.

### Sediment Depth Profiles

The sediments collected at all sites were blackish at depths below 4–10 cm, except for the sediments from station S3. The concentrations of DOC and FDOM in the porewaters increased with depth at stations S2, S4, S6, and S7, but this trend was not observed at the other stations (Figure 2). At all stations, the pH was lower in porewater than in the bottom water (Figure S1). Nitrate concentrations were higher in the porewater at 1–2 cm depth than in bottom water and porewater at 5–6 cm depth. DIC, ammonium, phosphorus, and silicate concentrations increased with the sediment depth. Sulfate concentrations were lowest in porewaters of 5–6 cm depth across all stations (Figure S1). At stations S1, S2, S6, and S7, the highest Mn^2+^ concentrations were measured at a depth of 1–2 cm, while the highest Fe^2+^ concentrations were usually at a depth of 5–6 cm. Fe^2+^ and Mn^2+^ were not detected in the porewaters of stations with coarse (S3) and medium sands (S5). Although aqueous Fe^2+^ was absent at a depth of 1–2 cm, abundant reduced Fe was extracted from the solid phase (Figure 2). The percentage of reduced Fe in poorly crystalline Fe (extractable with 0.5 M HCl) increased with depth. At depths of 5–6 cm, Fe(II) accounted for nearly 70% of labile Fe at stations S2 and S4, and more than 90% at stations S6 and S7 but less than 20% at stations S1 and S5.

The average molecular mass of DOM was very similar in bottom water and porewater at stations S1 and S2, varying around 363–367 Da (Table S4), while at stations S5, S6, and S7, the average molecular mass of DOM was higher in bottom water than in porewater. At S6 and S7, the average molecular mass of DOM at a depth of 5–6 cm, where nearly all labile Fe was present in the reduced form, was higher than that at a depth of 1–2 cm. More sulfur components were found in DOM from the porewater at a depth of 5–6 cm than in bottom water except at station S5. There was no distinguishable difference in aromaticity index (AI_mod_), H/C ratios, and O/C ratios between bottom water and porewater.

### DOM in Sediment Leachates

To quantitatively compare the distribution of DOM in the aqueous and solid phases, we normalized the abundance of DOM into per bulk of sediments (Table S2). We found that the abundance of DOM associated with the sedimentary solid phase was much higher than that in the porewater. DOM leached by UPW was about 54 ± 20 times more abundant than DOM in porewater. More DOM was leached out of all sediments by 0.5 M HCl than by UPW (1.7 ± 0.3 times), accompanied by the dissolution of poorly crystalline Fe oxyhydroxides and Fe monosulfides (Figure 2 and Table S2). The amount of DOM associated with poorly crystalline Fe was calculated as the difference between HCl and UPW leachate, and it was comparable to the amount of loosely adsorbed DOM (leached by UPW) at most stations (Table S2).

To compare DOM molecular compositions in the different leachates with bottom water and porewater, we conducted a PCoA with the molecular dataset (Figure S2). The two principal coordinates explained 68% of the DOM molecular composition. Three sample groups were distinguished, namely, DOM in bottom water/porewater, loosely sorbed DOM (UPW leachate), and loosely sorbed DOM plus Fe-associated DOM (HCl leachate). The variation between these groups was more obvious than the variation between different stations. Despite this, we found a positive correlation between *I*_Terr_ and salinity in the UPW leachates (Figure 3B). In addition, there was a negative correlation between the salinity and AI_mod_ (and *I*_DEG_) in HCl leachates (Figure 3C, D).

**Figure 4:**
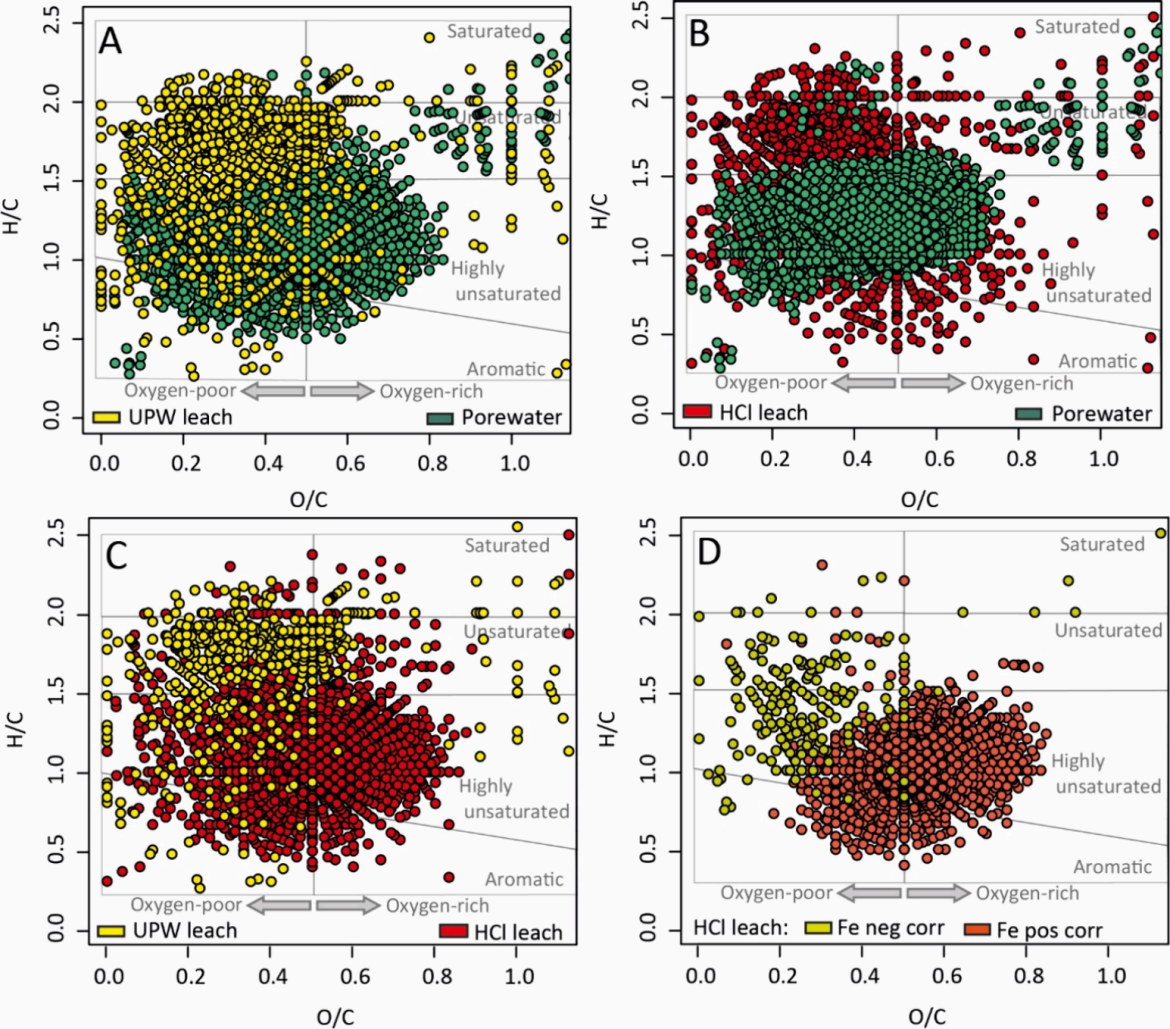
van Krevelen plots of selected DOM molecular formulas using their elemental O/C and H/C ratios. The plotted formulas in A–C were selected based on their relative abundances differing between two sample types as follows: (A) UPW leachate vs porewater (e.g., yellow symbols represent formulas higher in UPW leachate than porewater; green symbols represent formulas higher in porewater than UPW leachate); (B) HCl leachate vs porewater; (C) UPW leachate vs HCl leachate. (D) O/C and H/C ratios of molecular formulas significantly correlated with leached Fe from all sediments (e.g., Fe neg corr represents formulas significantly negatively correlated with leached Fe concentrations; Fe pos corr represents formulas significantly positively correlated with leached Fe concentrations).

The average molecular mass of DOM in porewater was higher than in the UPW and HCl leachates (Tables S4 and S5). DOM in the UPW leachate was more enriched in sulfur compared to all other sample types (bottomwater and porewater and HCl leachate). DOM in porewater had a higher O/C ratio and lower H/C ratio on average than DOM in the UPW and HCl leachates (Figure 4A, B). DOM in the HCl leachates had a higher O/C ratio and lower H/C ratio than those in the UPW leachate (Figure 4C). More aromatic compounds were identified in the HCl leachate than in the UPW leachate. In the HCl leachate, the relative proportions of oxygen-rich, aromatic, and highly unsaturated compounds were positively correlated with the concentration of leached Fe (Figure 4D).

## Discussion

### DOM Compositions along the Near- to OffShore Transect

Along our sampling transect, salinity, sulfate, and silicate, as well as *I*_Terr_ and FDOM, showed clear near- to offshore trends and a decrease of the terrigenous molecular signal in DOM (Figures 1–3). The proportion of aromatic compounds decreased, and that of unsaturated compounds increased from near- to offshore (Figure 3A). The negative correlations between salinity, *I*_Terr_, and FDOM indicated that the terrigenous signatures were attenuated mainly through conservative mixing with seawater.^24^ In general, the effects of ionic strength on DOM composition (e.g., coagulation and subsequent precipitation) were expected to be negligible due to the narrow salinity range in this study (30.1–34.6).^62−64^ The average molecular mass, O/C ratios, and *I*_DEG_ of DOM in bottom water increased from near- to offshore (Table S4), which is typical for the transition from land to ocean.^16,55^

Permeable sediments can filter massive amounts of seawater, thus potentially decreasing the terrigenous DOM signatures through adsorption (e.g., by Fe minerals in sediments) and degradation within the sediments.^12^ Indeed, we found higher *I*_Terr_ values in the UPW and HCl leachates than those in porewater and bottom water (Figure 3B), indicating an enrichment of terrigenous DOM associated with the solid phases. There was no significant correlation between DOM molecular compositions and sediment properties (e.g., average grain size, TOC). The expanded oxic conditions due to coarser grain sizes (e.g., station S3 and S5) or burrowing animals (e.g., station S1) did not cause distinguishable changes in DOM molecular compositions, indicating the limited effects of benthic DOM degradation and fluxes on molecular DOM signatures of bottom water.^65^ Therefore, we suggest that the trend of DOM composition in bottom water (from aromatic to highly unsaturated) was mainly caused by the conservative mixing of river water with seawater and to a lesser extent of DOM adsorption during bottom water filtration through the permeable sandy sediments.

### DOM Composition in the Subtidal Permeable Sediments

The studied region has high OM input from terrestrial sources (∼110 g C m^–2^ year^–1^) and marine primary production (309–430 g C m^–2^ year^–1^),^66^ and 10–20% of the primary production is remineralized in the sediments,^67^ making the permeable sediments hot spots for carbon cycling. The high *I*_DEG_ of DOM and the abundance of unsaturated compounds with average H/C ratios of 1.30 ± 0.04 and O/C ratios of 0.44 ± 0.02, as well as the low TOC contents and DOC concentrations measured in this study, indicate a high extent of DOM degradation and low OM burial rates in the sediment.^68,69^ Strong benthic–pelagic coupling and hydrological connectivity were indicated by the molecular similarity of DOM in bottom water and shallow porewater (1–2 cm) (Table S4).

For DOM in different depths of sediments, there was no significant trend in the shifting of H/C and O/C ratios like described in a previous study,^70^ perhaps due to the comparatively small intervals between the depths, and strong advective influence on the sediments investigated here. In deeper sediments (5–6 cm), the increased sulfur content of DOM in the porewater (Table S4), as well as the increased net sulfate consumption (Figure S1), point toward abiotic DOM sulfurization. Sulfurization likely increases the stability of DOM, thus potentially impeding DOM remineralization in sediments.^18,71−73^ Likewise, the sulfur content in the average formula of DOM increased in UPW and HCl leachates with increasing depths and was overall higher than in porewater (Table S5). In previous studies, contradictory results about selective sulfuric group enrichment in Fe-adsorbed DOM were reported.^45,74^ When sulfur was highly abundant (e.g., in a hydrothermal vent system), selective enrichment of sulfuric groups in Fe-DOM coagulates was found,^74^ while no such preferential coagulation of sulfuric DOM was observed during Fe coprecipitation experiments simulating subterranean estuaries.^45^ For the subtidal permeable sediments studied here, we suggest that the higher abundance of sulfuric groups in the solid phase is mainly caused by early diagenesis.^11,75−77^ Most likely, hydrogen sulfide originating from organoclastic sulfate reduction can react with Fe(II) in porewater and Fe(III) oxyhydroxides present in the solid phase to form the black precipitates (e.g., FeS) observed in our sediments.^43,75^ It is also consistent with our results that hydrogen sulfide was not detectable in the porewater, while net sulfate consumption occurred in the deeper sediment (Figure S1). Due to the low solubility of Fe monosulfides, the preferential reactions between hydrogen sulfide and Fe phases most likely hindered the abiotic sulfurization of DOM,^78^ which would explain the lower proportion of sulfur-containing molecular formulas in the HCl leachates than in the UPW leachates.

In our study, a similar amount of DOM was loosely adsorbed to the sediment (UPW leachates) as was associated with poorly crystalline iron (difference between HCl leachates and UPW leachates). Adsorbed DOM, often enriched along the edges and at discrete spots on mineral surfaces, may also have decreased bioavailability.^79,80^ Loosely adsorbed DOM (UPW leachate) had higher H/C and lower O/C ratios than DOM in porewater (Figure 4A), i.e., it was less oxidized, which is consistent with previous observations from experiments.^44^ However, contradictory to laboratory experiments,^44,81^ we did not observe the preferential adsorption of compounds with larger molecular masses by the solid phase. DOM in the UPW leachates was smaller in molecular mass than DOM in porewater. We note that the leaching procedures with UPW and HCl may cause the bursting of cells (due to osmotic shock) and release of low-molecular-mass metabolites and large biomolecules, which may not be recovered quantitatively via SPE.^82^ It could also partially explain the low DOM recoveries of leachate samples compared to bottom water and porewater samples.^82^

### DOM and Fe Interactions in Permeable Surface Sediments

The variable redox conditions in the permeable surface sediments caused by porewater advection enable Fe to play an especially important yet complex role in DOM turnover.^43,83^ As suggested in our previous study,^43^ Fe can function as a “redox battery” in the redox interface and repetitively serve as an electron acceptor for OM remineralization. Reactive oxygen species generated during Fe reoxidation could further alter DOM mineralization processes.^48^ In this study, we found that DOM associated with poorly crystalline Fe (extractable by 0.5 M HCl) was enriched in aromatic, oxygen-rich, and highly unsaturated compounds compared with porewater (Figure 4D). Our findings are consistent with previous adsorption or coagulation experiments using Fe oxyhydroxides.^18,45,81^ Such poorly crystalline Fe phases (e.g., ferrihydrite and mackinawite) could strongly affect DOM distribution in permeable surface sediments.

During reoxygenation caused by porewater advection from oxic zones, dissolved Fe(II) can be reoxidized and coprecipitated with DOM, and the formed Fe(III) oxyhydroxides can further adsorb DOM due to their relatively large surface area.^84,85^ The oxidative transformation of Fe monosulfides into Fe(III) oxyhydroxides may further enhance the DOM adsorption capacity of the solid phase.^83,85^ Under reducing conditions, DOM associated with Fe(III) oxyhydroxides may be remobilized due to microbial Fe reduction and release.^86^ Here, we did not observe strong positive correlations between DOC and Fe(II) concentrations in the porewater for most of the stations (Figure 2). There was also no observable change in DOM molecular composition when the Fe(II) concentration slightly increased in porewater (Figure 3 and Table S4). We suggest that frequent O_2_ intrusion into deeper sediments due to porewater advection could limit Fe(II) release from the sediments and accumulation in the porewater, which in turn impeded the release of Fe(III)-bound DOM. For deeper sediments with net sulfate consumption, the extensive Fe(III)-bound DOM release could be restricted via DOM resequestration by Fe monosulfides.^87^ Therefore, we suggest that poorly crystalline Fe phases in permeable surface sediments can serve as an intermediate storage for DOM. We further propose that Fe preferentially retains terrigenous DOM during seawater filtration through permeable sediments.^88^

In per volume of bulk sediment, the amount of DOM associated with poorly crystalline Fe phases was about 35 times the DOM in the porewater, meaning that it seems to be an important intermediate storage for DOM in the surface sediments. Further considering the preferential preservation of terrigenous OM by Fe, we suggest that the cycling of poorly crystalline Fe may play a big role in the source-to-sink processes of terrigenous OM and significantly affect their turnover rates. We further hypothesize that Fe phases in permeable sediments may facilitate OM burial due to their efficient sequestration of OM under dynamic redox conditions. Yet, considering the repetitive contributions of poorly crystalline Fe(III) oxyhydroxides to OM remineralization,^43^ as well as the possible molecular alterations by reactive oxygen species coproduced during Fe(II) oxidation,^48^ the overall effects of Fe on OM turnover are still hard to quantitively evaluate. Further investigations of the dynamic processes in permeable sediments are warranted to better understand carbon cycling in the coastal ocean.

## Supporting Information Available

Characterizations of studied stations; geochemical parameters of bottom water and porewater; DOM molecular characterizations; and other information (PDF)
