# Supplementary material for: Iron Promotes the Retention of Terrigenous Dissolved Organic Matter in Subtidal Permeable Sediments
Source: Environ Sci Technol. 2024 Apr 1;58(14):6204–14. doi: 10.1021/acs.est.3c09531 (PMC11008242; doi:10.1021/acs.est.3c09531)
Supplement: Supplementary file 1 — es3c09531_si_001.pdf [file es3c09531_si_001.pdf]

Iron promotes the retention of terrigenous dissolved organic matter in subtidal permeable sediments

Zhe Zhou,[1,2†*] Hannelore Waska,[3†] Susann Henkel,[1] Thorsten Dittmar,[3,4] Sabine Kasten,[1,5,6] Moritz Holtappels[1,5]

[1.] Alfred Wegener Institute Helmholtz Centre for Polar and Marine Research, 27570 Bremerhaven, Germany

[2.] State Key Laboratory of Marine Geology, Tongji University, 200092 Shanghai, China

[3.] Institute for Chemistry and Biology of the Marine Environment (ICBM), School of Mathematics and Science, Carl von Ossietzky Universität Oldenburg, 26129 Oldenburg, Germany

[4.] Helmholtz Institute for Functional Marine Biodiversity, University of Oldenburg, 26129 Oldenburg, Germany

[5.] MARUM - Center for Marine Environmental Sciences, University of Bremen, 28359 Bremen, Germany

[6.] Faculty of Geosciences, University of Bremen, 28359 Bremen, Germany

† *These authors contributed equally*

* *Corresponding author: zhe_research@outlook.com*

Supporting Information

Pages: 14 (including this page)

Tables: 7

Figures: 3

Method section: 1

Table S1. The characteristics of studied stations.

[a]Station NO.	Name	GPS	Water T (℃)	Depth (m)	Salinity (psu)	mean grain size (µm)	TOC (%)	% Silt	% Clay	% V Coarse Sand	% Coarse Sand	% Medium Sand	% Fine Sand	% V Fine Sand
1	Eider-1	54°16,38' N; 008°18,11' E	18	8.8	30.34	160.22	0.12±0.03	10.78	1.36	0.00	0.01	21.39	53.48	12.99
2	Eider-2	54°21,48' N; 008°08,96' E	18.4	12.3	30.15	151.80	0.12±0.05	12.70	2.32	0.00	0.01	20.59	56.91	7.45
3	Amrum Bank	54°37,10' N; 007°54,26' E	18	10	31.17	793.78	0.01±0.00	0.01	0.00	28.80	56.37	13.06	0.84	0.16
4	NOAH-E Nord	54°47,08' N; 007°37,72' E	18.1	16.2	31.86	216.21	0.04±0.01	2.14	0.70	0.00	0.02	31.96	61.21	3.95
5	Sylter Aussenriff	54°53,87' N; 006°54,18' E	17.3	25	33.02	307.61	0.01±0.00	0.91	0.51	0.00	4.89	69.68	21.11	2.90
6	NOAH-I Ost	55°27,66' N; 004°32,15' E	16.6	28	34.67	-	0.05±0.01	-	-	-	-	-	-	-
7	NOAH-I West	55°38,70' N; 003°40,44' E	16.5	36	34.64	188.84	0.05±0.01	2.93	0.83	0.00	0.00	19.17	68.82	8.10

[a.] The station number was assigned based on its distance to main estuary area (from closest to furthest).

Table S2. Parameters in the extractions of UPW and 0.5 M HCl, and their comparisons with porewater

Station	Depth	DOM in UPW leachate		DOM in HCl leachate		$DOM_{Fe\text{-}bound}$[b]		$DOM_{Fe\text{-}bound}$ / Fe_{total} in HCl leachate	DOM in porewater		$DOM_{UPW\ leachate}$/ $DOM_{porewater}$	$DOM_{Fe\text{-}bound}$/ $DOM_{porewater}$
	(cm)	(µmol C / g sand)	(µmol C / mL bulk)[a]	(µmol C / g sand)	(µmol C / mL bulk)	(µmol C / g sand)	(µmol C / mL bulk)		(µmol C / mL pore)	(µmol C / mL bulk)		
1	1-2	2.14±0.75	3.21±1.13	4.27±0.21	6.41±0.32	2.13±0.54	3.20±0.81	0.12	0.216	0.09	37.15	36.98
	5-6	2.89±1.93	4.34±2.90	3.99±0.73	5.99±1.10	1.95	2.93	0.14	0.26	0.10	41.68	28.13
2	1-2	3.43±0.29	5.15±0.44	5.93±0.05	8.90±0.08	2.50±0.34	3.75±0.51	0.16	0.278	0.11	46.27	33.72
	5-6	5.19±3.38	7.79±5.07	7.45±3.04	11.18±4.56	2.26±0.35	3.39±0.53	0.08	0.224	0.09	86.89	37.83
3	1-2	1.41±0.14	2.12±0.21	2.57±0.08	3.86±0.12	1.15±0.05	1.73±0.08	1.32	0.162	0.06	32.64	26.62
4	1-2	2.78±0.23	4.17±0.35	3.79±0.39	5.69±0.59	1.01±0.16	1.52±0.24	0.15	0.159	0.06	65.57	23.82
	5-6	2.14±0.26	3.21±0.39	3.77±0.18	5.66±0.27	1.63±0.08	2.45±0.12	0.21	0.187	0.07	42.91	32.69
5	1-2	1.19±0.09	1.79±0.14	2.20±0.18	3.30±0.27	1.01±0.09	1.52±0.14	0.84	0.115	0.05	38.80	32.93
	5-6	1.18±0.08	1.77±0.12	1.93±0.01	2.90±0.02	0.75±0.06	1.13±0.09	0.65	0.09	0.04	49.17	31.25
6	1-2	2.49±0.13	3.74±0.20	5.54±0.79	8.31±1.19	3.05±0.66	4.58±0.99	0.85	0.17	0.07	54.93	67.28
	5-6	2.43±0.02	3.65±0.03	4.40±0.16	6.60±0.24	1.96±0.17	2.94±0.26	0.67	0.25	0.10	36.45	29.40
7	1-2	2.48±0.12	3.72±0.18	3.68±0.11	5.52±0.17	1.20±0.23	1.80±0.35	0.22	0.108	0.04	86.11	41.67
	5-6	2.83±0.94	4.25±1.41	3.75±0.64	5.63±0.96	0.92±0.29	1.38±0.44	0.23	0.128	0.05	82.91	26.95

a. To quantitatively compare DOC in aqueous and solid phases, we normalized DOC abundance into per mL bulk sediment, assuming sands density as 2.5 g/mL and porosity as 0.4 in all stations.

b. $DOC_{Fe\text{-}bound}$ was calculated based on the difference of 0.5 HCl and UPW leachate, indicating DOC preserved by poorly crystalline Fe oxyhydroxides.

Table S3. Mn, P and Si in the extracted solution of 0.5 M HCl.

NO.	Depth (cm)	Mn (µmol /g sand)	Si (µmol /g sand)	P (µmol /g sand)
1	1-2	3.11±0.41	3.41±0.11	1.54±0.13
	5-6	3.75±1.94	3.10±0.87	1.20±0.38
2	1-2	3.50±0.02	3.33±0.08	1.72±0.46
	5-6	1.86±1.40	6.55±2.00	2.26±0.48
3	1-2	0.15±0.00	0.19±0.00	0.13±0.02
4	1-2	1.01±0.19	1.60±0.30	2.02±0.50
	5-6	0.15±0.02	1.80±0.22	1.71±0.58
5	1-2	0.30±0.02	0.30±0.00	1.44±0.38
	5-6	0.27±0.05	0.29±0.03	1.55±0.55
6	1-2	0.12±0.02	0.55±0.07	2.06±0.40
	5-6	0.04±0.00	0.77±0.04	2.14±0.05
7	1-2	0.28±0.05	0.82±0.01	2.33±0.25
	5-6	0.06±0.00	1.06±0.02	1.62±0.17

[a.] $C_{Fe\text{-}bound}$ was calculated based on the difference of 0.5 HCl and UPW leachate, indicating OC preserved by labile Fe phase.

Table S4. Molecular characterization of bottom water and porewater in studied stations.

Station NO.	Depth (cm)	Mean mass (Da)	Average formula	H/C ratio	O/C ratio	AI_{mod}	I_{DEG}	MLB_L	I_{Terr}
	>0	366.75	$C_{17.70}H_{22.44}O_{7.74}N_{0.38}S_{0.11}$	1.273	0.435	0.256	0.55	15.12	0.23
1	1-2	365.91	$C_{17.69}H_{22.17}O_{7.78}N_{0.36}S_{0.08}$	1.253	0.437	0.266	0.55	14.10	0.25
	5-6	362.77	$C_{17.35}H_{22.41}O_{7.68}N_{0.33}S_{0.16}$	1.315	0.440	0.242	0.56	16.16	0.24
	>0	364.91	$C_{17.83}H_{22.84}O_{7.62}N_{0.35}S_{0.06}$	1.278	0.428	0.258	0.52	20.78	0.24
2	1-2	365.82	$C_{17.58}H_{22.62}O_{7.76}N_{0.37}S_{0.11}$	1.304	0.441	0.248	0.56	19.64	0.25
	5-6	365.63	$C_{17.51}H_{22.61}O_{7.85}N_{0.35}S_{0.10}$	1.297	0.447	0.248	0.54	16.47	0.24
3	>0								
	1-2	373.79	$C_{18.07}H_{22.86}O_{7.99}N_{0.38}S_{0.05}$	1.264	0.444	0.257	0.54	18.39	0.20
	>0	369.08	$C_{18.03}H_{23.36}O_{7.71}N_{0.33}S_{0.05}$	1.296	0.430	0.248	0.48	21.38	0.20
4	1-2	375.57	$C_{18.23}H_{23.21}O_{7.96}N_{0.38}S_{0.04}$	1.269	0.438	0.256	0.57	18.31	0.19
	5-6	378.43	$C_{19.06}H_{26.74}O_{6.80}N_{0.53}S_{0.15}$	1.390	0.383	0.223	0.36	32.53	0.50
	>0	375.88	$C_{18.19}H_{23.07}O_{8.05}N_{0.35}S_{0.04}$	1.269	0.442	0.254	0.59	15.33	0.15
5	1-2	357.95	$C_{17.68}H_{23.15}O_{7.18}N_{0.34}S_{0.08}$	1.326	0.411	0.251	0.51	19.88	0.20
	5-6	358.95	$C_{18.61}H_{23.52}O_{8.29}N_{0.39}S_{0.04}$	1.266	0.445	0.254	0.65	14.70	0.15
	>0	388.64	$C_{18.67}H_{23.80}O_{8.44}N_{0.37}S_{0.03}$	1.275	0.452	0.245	0.64	15.26	0.14
6	1-2	365.68	$C_{17.47}H_{23.33}O_{7.74}N_{0.37}S_{0.13}$	1.374	0.444	0.218	0.60	20.88	0.15
	5-6	379.38	$C_{17.94}H_{24.26}O_{7.92}N_{0.55}S_{0.19}$	1.375	0.443	0.206	0.64	21.26	0.15
	>0	394.35	$C_{18.89}H_{24.06}O_{8.95}N_{0.40}S_{0.03}$	1.273	0.454	0.243	0.67	16.55	0.13
7	1-2	367.93	$C_{18.18}H_{24.14}O_{7.41}N_{0.36}S_{0.07}$	1.323	0.414	0.242	0.62	21.85	0.18
	5-6	374.65	$C_{17.80}H_{23.59}O_{8.00}N_{0.39}S_{0.14}$	1.367	0.450	0.217	0.65	19.79	0.13

Table S5. Molecular characterization of the DOM in different leachates.

NO.	Depth (cm)	UPW leachate							HCl leachate						
		Mean mass (Da)	Average formula	H/C ratio	O/C ratio	AI_{mod}	I_{DEG}	I_{Terr}	Mean mass (Da)	Average formula	H/C ratio	O/C ratio	AI_{mod}	I_{DEG}	I_{Terr}
1	1-2	329.40	$C_{16.79}H_{26.58}O_{5.54}N_{0.35}S_{0.19}$	1.563	0.349	0.151	0.17	0.54	342.39	$C_{16.99}H_{23.90}O_{6.63}N_{0.41}S_{0.07}$	1.402	0.398	0.214	0.32	0.51
	5-6	324.29	$C_{16.39}H_{25.45}O_{5.61}N_{0.26}S_{0.22}$	1.539	0.356	0.160	0.23	0.51	321.49	$C_{16.09}H_{21.84}O_{6.17}N_{0.31}S_{0.11}$	1.355	0.392	0.250	0.40	0.56
2	1-2	343.74	$C_{17.57}H_{27.22}O_{5.74}N_{0.47}S_{0.16}$	1.528	0.346	0.171	0.37	0.46	320.28	$C_{15.78}H_{21.85}O_{6.23}N_{0.33}S_{0.14}$	1.372	0.401	0.234	0.34	0.48
	5-6	353.70	$C_{17.72}H_{27.39}O_{6.29}N_{0.35}S_{0.18}$	1.529	0.373	0.159	0.23	0.51	338.19	$C_{16.50}H_{22.53}O_{6.76}N_{0.43}S_{0.11}$	1.365	0.417	0.234	0.39	0.57
3	1-2	342.74	$C_{18.41}H_{27.35}O_{5.05}N_{0.40}S_{0.18}$	1.488	0.308	0.207	0.28	0.52	318.83	$C_{16.45}H_{24.49}O_{5.44}N_{0.31}S_{0.14}$	1.481	0.344	0.196	0.31	0.51
4	1-2	329.88	$C_{17.33}H_{24.98}O_{5.17}N_{0.33}S_{0.23}$	1.450	0.332	0.226	0.31	0.60	298.10	$C_{15.01}H_{20.77}O_{5.61}N_{0.25}S_{0.12}$	1.393	0.379	0.237	0.33	0.52
	5-6	313.02	$C_{15.86}H_{24.24}O_{5.12}N_{0.54}S_{0.23}$	1.528	0.344	0.177	0.26	0.49	344.61	$C_{17.54}H_{27.49}O_{5.76}N_{0.64}S_{0.14}$	1.559	0.351	0.141	0.22	0.38
5	1-2	321.36	$C_{16.92}H_{26.27}O_{4.86}N_{0.41}S_{0.22}$	1.535	0.313	0.182	0.29	0.58	309.92	$C_{16.24}H_{24.30}O_{5.15}N_{0.27}S_{0.10}$	1.486	0.332	0.197	0.29	0.59
	5-6	332.00	$C_{17.29}H_{26.43}O_{5.14}N_{0.39}S_{0.24}$	1.522	0.324	0.186	0.39	0.50	312.06	$C_{16.45}H_{24.30}O_{5.14}N_{0.25}S_{0.09}$	1.467	0.331	0.210	0.30	0.60
6	1-2	338.54	$C_{17.38}H_{26.86}O_{564}N_{0.37}S_{0.18}$	1.528	0.350	0.179	0.28	0.63	298.37	$C_{15.11}H_{22.47}O_{5.24}N_{0.29}S_{0.18}$	1.478	0.367	0.198	0.28	0.61
	5-6	332.59	$C_{16.75}H_{26.01}O_{5.76}N_{0.41}S_{0.17}$	1.526	0.374	0.173	0.06	0.81	290.34	$C_{14.89}H_{22.27}O_{4.86}N_{0.34}S_{0.19}$	1.497	0.345	0.194	0.28	0.59
7	1-2	358.34	$C_{18.80}H_{29.15}O_{5.52}N_{0.43}S_{0.23}$	1.533	0.327	0.177	0.31	0.60	305.38	$C_{15.63}H_{22.66}O_{5.49}N_{0.23}S_{0.12}$	1.444	0.363	0.213	0.34	0.47
	5-6	340.38	$C_{17.74}H_{28.11}O_{5.06}N_{0.51}S_{0.30}$	1.572	0.314	0.154	0.30	0.59	314.14	$C_{15.92}H_{23.44}O_{5.49}N_{0.36}S_{0.21}$	1.478	0.361	0.190	0.31	0.48

Table S6. The abundance of DOM compounds in offshore and nearshore stations.

Compound group	Offshore (n)	Offshore (%)	Nearshore (n)	Nearshore (%)
Aromatic	6	1	367	28
Highly unsaturated	798	90	794	61
Unsaturated	78	9	143	11
Saturated	5	1	0	0

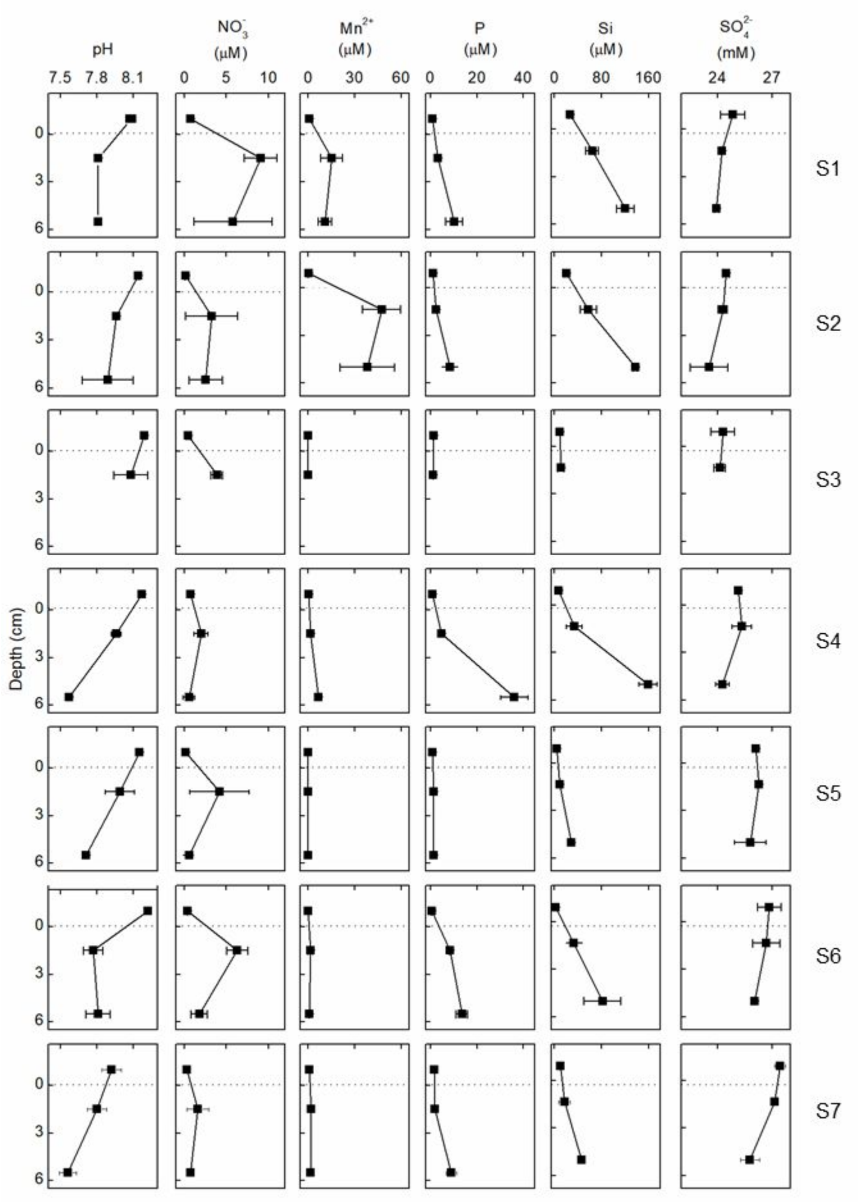

Figure S1. Inorganic parameters of bottom water and porewater (1-2 cm and 5-6 cm) in studied stations. Duplicate cores/samples were collected and measured for each station. Detailed numbers can be found in Table S2.

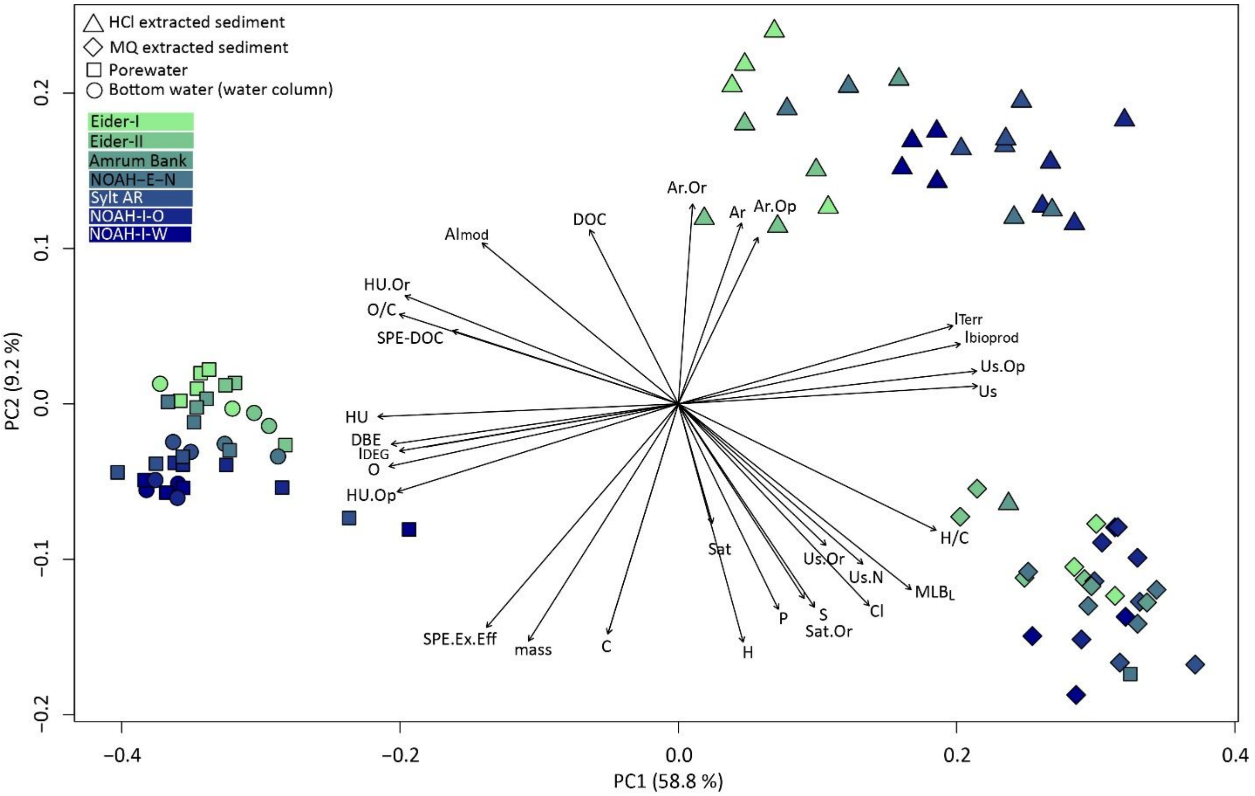

Figure S2. A principal coordinate analysis (PCoA) of measured samples. Symbols indicate different sample origins and treatments. Green to dark blue colors indicate the land-ocean trend. Ar = Aromatic, H = Highly unsaturated, Us = Unsaturated, Us.N = Unsaturated containing N, Sat = Saturated. Or = oxygen-rich, Op = oxygen-poor. DBE = double bond equivalents. C, H, S, P, Cl indicate relative abundances of elements in molecular formulae. SPE.Ex.Eff = SPE extraction efficiency.

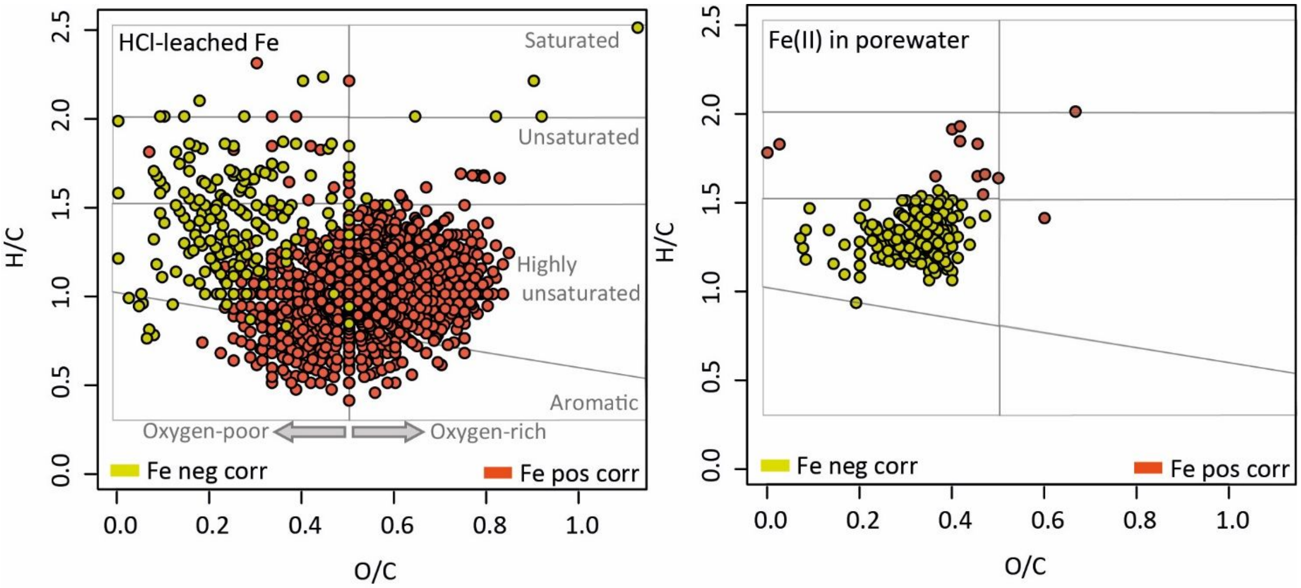

Figure S3. Comparison of molecular formulae associated with HCl-leached Fe from all sediments and the released Fe(II) in porewater. The results are derived from correlations between compound signal intensities and leached Fe concentrations, or Fe(II) concentration measured in porewater (Spearman's rank test, $p < 0.05$, $\rho > 0.5$ and -0.5 for positive and negative correlations, respectively).

The method of FT-ICR-MS measurement

After SPE, the methanol extracts were adjusted to a DOC concentration of 2.5 ppm with 1:1 methanol : ultrapure water, filtered through 0.2 μm PTFE filters, and analyzed on a 15 Tesla Fourier transform ion cyclotron resonance mass spectrometer (FT-ICR-MS, Bruker solariX XR), equipped with an electrospray ionization (ESI) source and a HyStar Autoanalyzer. A first measurement series revealed strong interferences in approximately one-third of the samples due to Na-Cl clusters. Therefore, all samples were dried down to remove the methanol, picked up in ultrapure water adjusted to pH=2 (HCl), re-extracted and re-measured. FT-ICR-MS measurement settings were broadband ESI negative ionization mode, with a flow rate of 40 $\mu L\ min^{-1}$, an ion accumulation time of 0.5-1 s, and 200 scans acquired. Each sample was injected twice, resulting in two analytical replicates. An in-house deep-sea reference sample (North Equatorial Pacific Intermediate Water, NEqPIW) was measured every ten injections to control instrument drift.

After measurements, all mass spectra were calibrated with an error of <0.1 ppm using a mass list based on ubiquitous compounds in NEqPIW. The calibrated mass spectra were extracted from the Bruker software with a VBS script and uploaded into the ICBM-OCEAN data processing pipeline to remove instrument noise, align matching masses, and assign unique molecular formulae to each mass with an error of <0.5 ppm and with the elemental setting $C_{1-100}H_{1-200}O_{1-70}N_{0-6}S_{0-2}P_{0-1}Cl_{0-1}$.[1] Together with a sample crosstable and molecular formulae information, the ICBM-OCEAN pipeline provides calculated compound group information (for example, aromatic, unsaturated, and saturated compound class) and other molecular indices such as double-bond equivalents (DBE) and the aromaticity index (AI.mod),[2] which are primarily based on elemental stoichiometries. Note that while the original mass lists derived from FT-ICR-MS measurements provide m/z (mass-over-charge ratios), ICBM-OCEAN only considers singly ionized compounds. Therefore, the m/z

values remaining after molecular formula assignment are equal to mass (in Da) and will be referred to as such for the discussion of results.

The resulting data crosstable was pre-processed in MS Excel for later statistical analyses. First, all sample mass spectra were blank-corrected using the SPE process blanks. All masses which had an occurrence of n≥2 in the process blank samples, and for which the average single intensities of the process blanks were higher than the average signal intensities of all samples, were removed from the dataset. In addition, all ^{13}C-, ^{18}O-, ^{15}N-, ^{34}S-, and ^{37}Cl-isotopologue containing molecular formulae were filtered out to reduce multiple influences of masses with high signal intensities. Finally, all analytical replicates were combined into a single mass spectrum for each sample, which only contained masses occurring in both replicates, and which had average signal intensities from the two analyses for each remaining mass.

The data crosstable was extracted as .csv file and further processed with R studio (R version 3.5.3), using mainly R base functions as well as the packages *vegan*, *corrplot*, and *plyr*. At the start of the data analysis, all sample mass spectra were normalized to a sum of 1 each. Using R, environmental indices such as the molecular lability boundary index (MLB_L),[3] the degradation index (I_{DEG}),[4] the bio-productivity index ($I_{bioprod}$),[5] the terrestrial index (I_{Terr}),[6] and the aromatic index (AImod),[2] were calculated for each sample (Table S6).[7]

Table S7. Molecular indices calculated from DOM molecular data. The AI_{mod} is calculated for each assigned molecular formula using the numbers of the elements *CHONSP*.[2] The MLB_L is calculated for each sample using the percent abundance of pre-defined H/C ratios for the entirety of assigned molecular formulae.[3] The I_{DEG} is calculated for each sample using a pre-defined set of ten assigned formulae which are either negatively (NEG_{IDEG}) or positively (POS_{IDEG}) correlated with $\Delta^{14}C$ radiocarbon ages along a depth gradient in the Atlantic Ocean.[4] The $I_{bioprod}$ is calculated for each sample using a pre-defined set of ten assigned formulae which are either positively (*B1-B5*) or negatively (*D1-D5*) correlated with DOM bioproduction by phytoplankton in a controlled laboratory mesocosm.[5] The I_{Terr} is calculated for each sample using a pre-defined set of eighty assigned formulae which are either negatively (*Terr*) or positively (*Mar*) correlated with salinity along the Amazon estuary.[6]

**Full Name**	**Short name**	**Equation**	**Reference**
Aromaticity index	AI_{mod}	$AI_{mod} = \frac{1 + C - 0.5O - S - 0.5(N + P + H)}{C - 0.5O - N - S - P}$	2
Molecular lability boundary	MLB_L	$MLB_L = 100 \times \left(\frac{(\#\ of\ molecular\ formulas\ with\ H/C \geq 1.5)}{(total\ \#\ of\ molecular\ formulas)}\right)$	3
Degradation index	I_{DEG}	$I_{DEG} = \left(\frac{\sum(magnitudes\ NEG_{IDEG})}{\sum(magnitudes\ NEG_{IDEG} +\ POS_{IDEG})}\right)$	4
Bioproduction index	$I_{bioprod}$	$I_{bioprod} = \frac{(B1 + B2 + B3 + B4 + B5)}{(D1 + D2 + D3 + D4 + D5)}$	5
Terrestrial index	I_{Terr}	$I_{Terr} = \left(\frac{\sum(magnitudes\ Terr)}{\sum(magnitudes\ Terr +\ Mar)}\right)$	6

References

1. Merder, J.; Freund, J. A.; Feudel, U.; Hansen, C. T.; Hawkes, J. A.; Jacob, B.; Klaproth, K.; Niggemann, J.; Noriega-Ortega, B. E.; Osterholz, H.; Rossel, P. E.; Seidel, M.; Singer, G.; Stubbins, A.; Waska, H.; Dittmar, T., ICBM-OCEAN: Processing Ultrahigh-Resolution Mass Spectrometry Data of Complex Molecular Mixtures. *Analytical Chemistry* **2020,** *92* (10), 6832-6838.
2. Koch, B. P.; Dittmar, T., From mass to structure: An aromaticity index for high-resolution mass data of natural organic matter. *Rapid communications in mass spectrometry* **2006,** *20* (5), 926-932.
3. D'Andrilli, J.; Cooper, W. T.; Foreman, C. M.; Marshall, A. G., An ultrahigh-resolution mass spectrometry index to estimate natural organic matter lability. *Rapid Communications in Mass Spectrometry* **2015,** *29* (24), 2385-2401.
4. Flerus, R.; Lechtenfeld, O. J.; Koch, B. P.; McCallister, S. L.; Schmitt-Kopplin, P.; Benner, R.; Kaiser, K.; Kattner, G., A molecular perspective on the ageing of marine dissolved organic matter. *Biogeosciences* **2012,** *9* (6), 1935-1955.
5. Seibt, M. The molecular geography of dissolved organic matter in the Atlantic and Southern Ocean. Universität Oldenburg, 2017.
6. Medeiros, P. M.; Seidel, M.; Niggemann, J.; Spencer, R. G. M.; Hernes, P. J.; Yager, P. L.; Miller, W. L.; Dittmar, T.; Hansell, D. A., A novel molecular approach for tracing terrigenous dissolved organic matter into the deep ocean. *Global Biogeochemical Cycles* **2016,** *30* (5), 689-699.
7. Waska, H.; Simon, H.; Ahmerkamp, S.; Greskowiak, J.; Ahrens, J.; Seibert, S. L.; Schwalfenberg, K.; Zielinski, O.; Dittmar, T., Molecular Traits of Dissolved Organic Matter in the Subterranean Estuary of a High-Energy Beach: Indications of Sources and Sinks. *Frontiers in Marine Science* **2021,** *8*.
